# Antioxidant Activity of Novel Casein-Derived Peptides with Microbial Proteases as Characterized via Keap1-Nrf2 Pathway in HepG2 Cells

**DOI:** 10.4014/jmb.2104.04013

**Published:** 2021-06-28

**Authors:** Xiao Zhao, Ya-Juan Cui, Sha-Sha Bai, Zhi-Jie Yang, Miao-Cai, Sarah Megrous, Tariq Aziz, Abid Sarwar, Dong Li, Zhen-Nai Yang

**Affiliations:** 1 Beijing Advanced Innovation Center for Food Nutrition and Human Health, Beijing Engineering and Technology Research Center of Food Additives, Beijing Technology and Business University, Beijing 100048, P.R. China; 2 Beijing Institute of Nutrition Resources, Beijing 100069, P.R. China

**Keywords:** Microbial protease, casein hydrolysate, antioxidant peptide, Keap1-Nrf2 pathway, HepG2 cells

## Abstract

Casein-derived antioxidant peptides by using microbial proteases have gained increasing attention. Combination of two microbial proteases, Protin SD-NY10 and Protease A “Amano” 2SD, was employed to hydrolyze casein to obtain potential antioxidant peptides that were identified by LCMS/ MS, chemically synthesized and characterized in a oxidatively damaged HepG2 cell model. Four peptides, YQLD, FSDIPNPIGSEN, FSDIPNPIGSE, YFYP were found to possess high 1,1-diphenyl-2-picrylhydrazyl (DPPH) scavenging ability. Evaluation with HepG2 cells showed that the 4 peptides at low concentrations (< 1.0 mg/ml) protected the cells against oxidative damage. The 4 peptides exhibited different levels of antioxidant activity by stimulating mRNA and protein expression of the antioxidant enzymes such as superoxide dismutase (SOD), catalase (CAT) and glutathione peroxidase (GSH-Px), as well as nuclear factor erythroid-2-related factor 2 (Nrf2), but decreasing the mRNA expression of Kelch-like ECH-associated protein 1 (Keap1). Furthermore, these peptides decreased production of reactive oxygen species (ROS) and malondialdehyde (MDA), but increased glutathione (GSH) production in HepG2 cells. Therefore, the 4 casein-derived peptides obtained by using microbial proteases exhibited different antioxidant activity by activating the Keap1-Nrf2 signaling pathway, and they could serve as potential antioxidant agents in functional foods or pharmaceutic preparation.

## Introduction

Casein, the main component of bovine milk, accounts for about 80% of the total milk protein, which not only provides abundant amino acids for the body, but is also the main source of bioactive peptides [1]. The bioactive peptides from casein that are present in inactive form as part of casein sequence can be released to become physiologically active by hydrolysis with appropriate proteases [2]. A large number of bioactive peptides were obtained for application in food and medicine industry by in vitro enzymatic hydrolysis under optimized conditions [3]. Various bioactive peptides possessing physiological functions in the cardiovascular, endocrine, immune, and nervous systemsfrom casein could be obtained by hydrolysis with different enzymes [4]. Among the bioactive peptides from casein, antioxidant peptides have gained increasing attention due to their capabilities of removing excessive free radicals, inhibiting lipoxygenase activity, decomposing oxides, and enhancing anti-aging and disease resistance in human body [5, 6].

Hydrolysis of casein by the use of extracellular proteases from microbes is advantagerous since these enzymes can be produced by microbial fermentation with relatively high yield but low cost, and with wide variety of microbial sources, when compared with animal and plant-derived proteases [7]. Among the microbial proteases, Protin SD-NY10 (P-NY10) is an end-type neutral metalloprotease extracted from *Bacillus amyloliticus* that hydrolyzes peptide bonds between hydrophobic amino acid residues [8]. Another Protease A “Amano” 2SD (P-2SD) is a neutral protease derived from fungus with high exo- and endo-peptidase activities [9]. P-NY10 was shown to hydrolyze soya milk to increase its ACE inhibitory activity [8]. Combined use of P-2SD and the protease of Protex 6L was shown to hydrolyze soybean protein to increase its antioxidant activity with good sensory properties [10]. Although P-NY10 and P-2SD were proved to be safe and productive for application in the food industry, these two enzymes had not been used for hydrolysis of casein to produce bioactive peptides including antioxidant ones.

Kelch-like ECH-associated protein 1 (Keap1) and nuclear factor erythroid-2-related factor 2 (Nrf2) constitute the important signal pathway regulating the antioxidant system to maintain the redox balance and metabolism of the cell by adjusting 1% to 10% of the genes [11]. Activation of the Keap1-Nrf2 signaling pathway results in expression and transcriptional regulation of some important proteins and enzymes in the body, such as glutathione peroxidase, glutathione S-transferase, superoxide dismutase, heme oxidase, catalase, thioredoxin reductase, etc., which exert detoxification and antioxidant defense functions. The Keap1-Nrf2 signaling pathway has been shown to involve in a variety of diseases related to oxidative stress, including cancer, Alzheimer's disease, Parkinson's disease, diabetes, etc. [12, 13]. Therefore, discovering and studying the activators of Keap1-Nrf2 signaling pathway is of great significance for the prevention and treatment of diseases induced by oxidative stress.

To explore casein-derived antioxidant peptides, the present study was carried out to hydrolyze casein by using two microbial proteases, P-NY10 and P-2SD at different ratios. The hydrolysates were fractionated by membrane filtration to obtain peptide fractions that were identified by liquid chromatography-tandem mass spectrometry (LC-MS/MS). Peptides with potential antioxidant activity were chemically synthesized and characterized through the Keap1-Nrf2 signaling pathway in a HepG2 cell model that was oxidatively damaged by AAPH (2, 2-azobis (2-methylpropylimid) dihydrochloride). The effects of the peptides on the cell viability, protein and gene expression, as well as activities of the antioxidant enzymes in HepG2 cells were studied to elucidate the mechanism of antioxidant action of the peptides.

## Materials and Methods

### Materials

Casein powder (90%, w/w) for preparing peptides was purchased from Fonterra Co-operative Group (Auckland, New Zealand). The commercial Protin SD-NY10 (8.38 × 10^4^ U/g, optimum temperature 45-50°C, optimum pH 7.0-8.0) and Protease A “Amano” 2SD (14.4 × 10^4^ U/g, optimum temperature 45-50°C, optimum pH 6.0-pH 9.0 for hydrolysis of casein were provided by Amano Enzyme Inc. (Japan). The monomer peptides were synthesized and provided by APeptide Co., Ltd. (China). Acetonitrile (ACN), methanol and trifluoroacetic acid were HPLC grades. All other reagents were analytically grade purchased from Banxia Biotechnology Co., LTD (China).

Human hepatoma (HepG2) cells were purchased from Peking Union Medical College (China). AAPH was purchased from Sigma–Aldrich (USA). Cell-counting kit-8 (CCK-8) was purchased from Dojindo (Japan). Dulbeccós modified eagle medium (DMEM), penicillin, streptomycin, 0.25% of trypsin were purchased from Gibco Life Technologies (USA). Radio Immunoprecipitation Assay (RIPA), Bicinchoninic acid (BCA) were purchased from Thermo Scientific, USA. Bovine serum albumin (BSA), Tween-20 (TBST) were purchased from (Amresco Inc., USA). Goat anti-mouse/rabbit IgG (HRP) was purchased from Kangwei Biological Company, Beijing, China. All assay kits of reactive oxygen species (ROS), malondialdehyde (MDA), glutathione (GSH), glutathione peroxidase (GSH-Px), catalase (CAT), superoxide dismutase (SOD), were purchased from Nanjing Jiancheng Bioengineering Institute (China). Primary monoclonal antibodies of Anti-CAT (GTX110704), anti-SOD (Ab13533), anti-GSH-Px (Ab22604), anti-Nrf2 (Ab31163), anti-Keap1 (Ab218815) and Anti-glyceraldehyde-3-phosphate dehydrogenase (GAPDH, AB-P-R001) were purchased from Abcam (UK).

### Preparation and Fractionation of Casein Hydrolysates

Two microbial proteases, P-NY10 and P-2SD in different ratios: 6:0, 5:1, 4:2, 3:3, 2:4, 1:5, 0:6, were added to 10%casein solution (10 g casein dissolved in 100 mL water ) at the total protease concentration of 0.5% (w/v). The mixture was mixed and kept at 50°C and pH 7.0, which was adjusted every 10 minutes using a FE20 pH meter (Mettler-Toledo). After hydrolysis for 6 h, the hydrolysates were kept in water bath at 90°C for 5 min to inactivate the proteases, then DH and DPPH scavenging ability of the hydrolysates were determined according to the above methods.

The best ratio between the two proteases to obtain proper DH and high DPPH scavenging ability of the hydrolysate was selected for preparation of casein hydrolysates for this study. 10% casein solution was hydrolyzed with the combination of proteases at 0.5% (w/v) at 50°C, pH 7.0 for 6 h, then the hydrolysate was centrifuged at 6,000 rpm, 4°C for 15 min. The peptides in the supernatant were separated into different fractions by membrane filtration using filters with different molecular weight cut off (1 kDa, 3 kDa, and 5 kDa) (Millipore Co., USA). DPPH clearance ability of permeate and retentate from each step were assayed. Fractions with high antioxidant capacity were collected and freeze-dried for further use.

### Identification of Peptides by LC-MS/MS

Peptides were separated by high-performance liquid chromatography with a C_18_ column (3 um, 100 A)(Agilent Technologies Inc., USA) at flow rate of 300 nL/min. The mobile phases include solution A, consisting of 0.1% formic acid in ultrapure water, and solution B, consisting of 0.1% formic acid in 80% ACN. The separation procedure was as follows: solution B was kept at 5% for 5 min, increased from 5% to 50% in 20 min, from 50% to 90% in 5 min, kept at 90% for 5 min, then decreased to 5% in 10 min. The separated peptides entered the Q Exactive mass spectrometer (Thermo fisher, USA) directly for online detection. Parameters for the primary mass spectrometry were: resolution, 70,000; AGC target, 3 × 10^6^; maximum IT, 40 ms; scan range, 350 to 1,800 m/z. Parameters for the second-stage mass spectrometry were: resolution, 17,500; AGC target, 1 × 10^5^; maximum IT, 60 ms; topN, 20; NCE / stepped NCE, 27. MaxQuant (version 1.6.1.0) was used for data retrieval. The retrieval parameters were as follows: Protein FDR was 0.01; The minimum amino acid length was set as 3; Variable modifications were N-Acetyl and Oxidation (M); Fixed modificationsis were Carbamidomethyl (C); The enzyme settings were selected as trypsin and others; Peptide mass tolerance was 20 ppm; Fragment mass tolerance was 0.6 Da; Mass values were set as monoisotopic; Significance threshold was 0.05. The peptide structure was characterized by matching and explaining the tandem mass spectra using similar strategy as previously reported [14], and the reliability of protein identification was determined by the high scores of proteins and peptides in the search results.

### Determination of DPPH Scavenging Ability

The extracellular antioxidant activity was determined by DPPH scavenging ability tests according to the method reported earlier [15]. Briefly, 1 ml of the peptide sample solution and 2 ml of 0.2 mmol/l DPPH methanol solution were mixed evenly, and reacted for 1 h at room temperature in the dark. Then the mixture was centrifuged (8 000 ×*g*, 4°C, 10 min), and the supernatant was taken for measurement of absorbance at 517 nm. The blank was prepared by replacing the DPPH solution with an equal volume of methanol, and the control was prepared by replacing the DPPH solution with an equal volume of sterile water. The calculation formula of DPPH free radical scavenging rate is:

Clearance rate (%) = [1−(A_1_−A)/A_0_] × 100

In the formula: A is the absorbance of the blank group; A_0_ is the absorbance of the control; A_1_ is the absorbance of the samples.

### Degree of Hydrolysis (DH)

The degree of hydrolysis was analyzed by the O-phthalaldehyde (OPA) method [16] with some modifications. Briefly, OPA reagent was prepared by dissolving 7.620 g sodium tetraborate (CAS 1303-96-4) and 200 mg sodium dodecyl sulfate (SDS) in 150 ml of deionized water. Then 160 mg O-phthalaldehyde (OPA, purity 97%) dissolved in 4 ml absolute ethanol was added, followed by addition of 176 mg 1,4-dimercaptothreitol (DTT, purity 99%), and filling deionized water till total volume of 200 ml. Each 400 μl sample solution was mixed with 3 mLOPA reagent, and the absorbance was measured at 340 nm by a U-3900 Spectrophotometer (Hitachi, Japan). The calculation formula is as follows: DH (%) = h/h_tot_×100%, where h_tot_ is the total number of peptide bonds per gram of protein and h is the total number of peptide bonds per gram of hydrolyzed protein, and h = (SerineNH2 − β)/α, where α, β, and h_tot_ are 1.039, 0.383, and 8.2 mEq/g protein in casein, respectively.

### Determination of Cell Viability by CCK-8

HepG2 cells were cultured in DMEM containing 10% of fetal bovine serum (FBS), 50 μg/ml of each penicillin and streptomycin at 37°C in a humidified incubator with 5% CO_2_. Subsequently, the HepG2 cells were digested with 0.25% trypsin and counted, and spread to a 96-well plate with the cell number of 1×10^5^/mL. DMEM containing different concentrations of peptides (0.1, 0.5, 1.0, 5.0, 10 mg/ml), or AAPH (25, 50, 100, 200, 400, 800, 1,500, 2,000 μmol/l) was then added, and the cells were cultured for 24 h under conditions of 95% air and 5% CO2 at 37°C. The cell viability was determined by adding 10 μl of CCK-8 solution to each well and mixing gently. After incubation at 37°C for 3 h in the dark, the OD value was measured at 450 nm.

### Gene Expression by Real-time Quantitative PCR

HepG2 cells were pre-treated with peptides at concentrations of 1.0 mg/ml for 4 h, and subsequently incubated in the presence of 200 μmol/l of AAPH for 18 h. Cells treated with DMEM were used as control group.

The relative changes in gene expression of SOD, CAT, GSH-Px, Nrf2 and Keap1 were examined by RT-PCR and analyzed by the ΔΔCT method, using GAPDH as an internal standard. Primers listed in Table 1 were designed and synthesized based on gene sequences in NCBI database. The total RNA of HepG2 cells was extracted using TRIzol reagent (Life Technologies, USA) according to the instructions. The RNA was reverse-transcribed with a M-MLV reverse transcription kit (Promega Corporation, USA) according to the instructions. A CFX96 Touch RT-qPCR system (Bio-Rad, USA) was used to assay mRNA expression. The amplification procedure of RT-qPCR was 1 min at 95°C for 1 cycle, 15 sec at 95°C and 50 sec at 60°C for 40 cycles.

### Western Blot Analysis

HepG2 cells were pre-treated with different concentrations of each peptide (0.1, 0.5, 1.0 mg/ml) for 4 h, and subsequently incubated in the presence of 200 μmol/l of AAPH for 18h. Then 200 μl of RIPA cell lysate (Thermo Scientific, USA) was added to the cell pellet to resuspend the cells, placed on ice for 30 min, and centrifuged for 15 min at 4°C, 12,000 rpm/min. After centrifugation, the supernatant was taken to be tested. The final protein concentration was determined by BCA protein assay kit (Thermo Scientific), then boiled in 5 × loading buffer for 10 minutes, and cooled down. The electrophoresis was performed on sodium dodecyl sulfate-polyacrylamide gel (SDS-PAGE) with 10% separating gel and 5% concentrated gel. The protein loading was around 30 μg. The 5%concentrated gel was kept at a constant pressure of 90V for about 20 min. The 10% separation gel was kept at a constant pressure of 160 V, and the electrophoresis stop time was determined by the pre-staining protein marker. After that, proteins were transferred onto an NC membrane (Millipore), submerged in 5% BSA-TBST, and shaked gently at room temperature for 30 min. Then the membranes were incubated with primary antibody, diluted with 1% BSA-TBST, kept at room temperature for 10 min, and placed at 4°C overnight. After the membrane was washed for 5 times with TBST continuously, it was incubated with the secondary antibody of goat anti-mouse/rabbit IgG (H+L) HRP, diluted with 1% BSA-TBST, then shaked gently at room temperature for 50 min. The membrane was washed with TBST for 6 times. Finally ECL was added to the film to react for 3-5 min, followed by film exposure.

### Assay of Intracellular Biochemical Indices

Quantification of ROS, MDA and GSH was performed by ROS, MDA and GSH assay kits according to the kit instructions. Activities of the enzymes associated with antioxidation were determined by CAT, GSH, SOD and GSH-Px assay kits according to the kit instructions.

### Statistical Analysis

All experiments were performed in triplicate. and the results were presented as the mean ± standard deviation. All data were analyzed by SPSS version 17.0 (SPSS, Inc., USA). Significant differences between treatments were determined by the level of P values less than 0.05 using ANOVA.

## Results and Discussion

### Hydrolysis of Casein by P-NY10 and P-2SD at Different Ratios

**Fig. 1:**
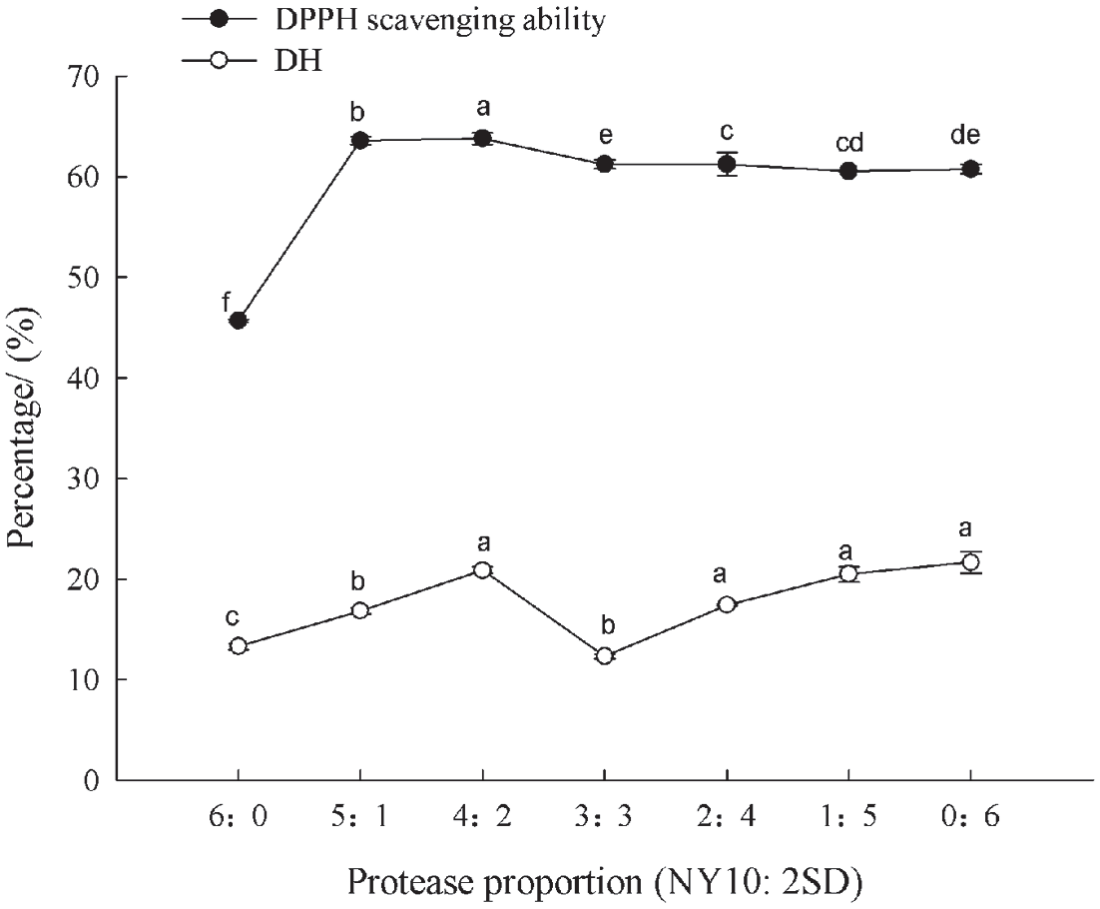
DPPH scavenging ability and hydrolysis degrees (DH) of the casein hydrolysates by different ratios of Protin SD-NY10 and Protease A “Amano” 2SD. Data are presented as mean ± SD, and results marked with the same letters are not significantly different (*p* > 0.05).

As shown in Fig. 1, the hydrolysis degree of casein by P-NY10 alone was about 13%, and reached the highest value (about 22%) when more P-2SD was added to the P-NY10 and P-2SD ratio of 4:2, corresponding to the DPPH scavenging ability at 63.80% of the hydrolysate. However, further addition of P-2SD to increase its proportion between the two proteases decreased the DPPH scavenging ability of the hydrolysate though there were changes of hydrolysis degrees. Therefore, higher antioxidant activity of the casein hydrolysate could be obtained by combined use of P-NY10 and P-2SD than that of either of them alone. Similarly, high antioxidant activity of goat milk casein hydrolysates was obtained by combined use of neutral and alkaline proteases [17]. However, the radical scavenging activity of bovine casein hydrolysate (13.1%) prepared with combination of trypsin and pepsin did not increase obviously when compared with that using trypsin alone (13.7%) [18]. Thus, bioactivities of casein hydrolysates may vary with the proteases used either alone or by combination of them mainly due to the difference in their cleavage sites on casein, resulting in hydrolysates with different peptide composition even at the same hydrolysis degree. Selection and appropriate combination of proteases are necessary for obtaining casein hydrolysate with higher bioactivity.

### Antioxidant Activity of the Separated Peptide Fractions

**Fig. 2:**
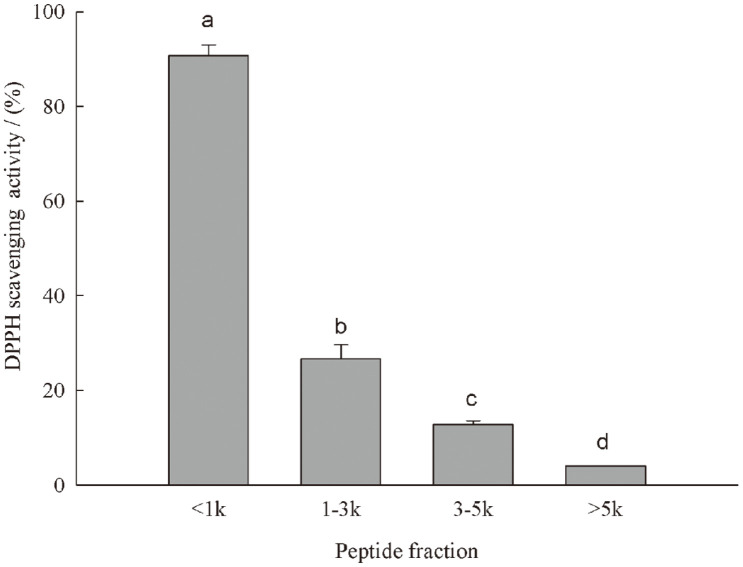
DPPH scavenging ability of different peptide fractions. Data are presented as mean ± SD, and results marked with the same letters are not significantly different (*p* > 0.05).

The casein hydrolysate obtained with P-NY10 and P-2SD (4:2) was subject to membrane separation, resulting in four peptide fractions (<1 kDa, 1-3 kDa, 3-5 kDa, >5 kDa). As shown in Fig. 2, the DPPH scavenging ability (94.72%) of the low-molecular-weight peptide fraction (< 1 kDa) was significantly (*p* < 0.05) higher than those of higher-molecular-weight fractions (< 26.67%). Antioxidant activity of the bovine casein hydrolysate containing high amount of peptides less than 1 kDa was also reported earlier [19]. High antioxidant activity was reported for the buffalo casein hydrolysate with molecular weights less than 1.5 kDa [20]. Generally, peptides less than 1 kDa exhibited high radical scavenging activity due to the combined effect of their scavenging of active oxygen and hydrogen atoms donation [18, 21].

Previously, DPPH scavenging ability (53.75%) and metal chelating capacity (97.74%) of the casein hydrolysate achieved by a new fungal protease isolated from *Myceliophthora thermophila* were obtained with the best antioxidant activity when compared with the hydrolysates from egg and whey proteins [22]. Likewise, hydrolysates with good antioxidant activity were obained from camel milk caseins using alcalase, α-chymotrypsin, and papain [23]. Rossini *et al*. (2009) also showed that casein hydrolysate by flavourzyme exhibited greater antioxidant properties for containing higher concentration of free amino acids and low molecular weight peptides [24]. Therefore, the antioxidant properties of the casein hydrolysates were strongly influenced by the affinity and specificity of enzymes for substrates, and higher antioxidant activity of casein hydrolysates could be obtained by using selected specific proteases as shown in this study.

### Identification of Potential Antioxidant Peptides

The structure and sequence of peptides could be analyzed on the basis of the mass to charge ratio (m/z) of the molecular ion peak as described earlier [25], and the result was confirmed by comparison with the data from the National Center for Biotechnology Information (NCBI) database. In this study, identification of the low-molecular-weight peptide fraction (< 1 kDa) with high antioxidant activity by LC-MS/MS demonstrated a total of 77 peptides. Among them, nine peptides mostly originated from α_s1_-casein with greater intensity were listed in Table 2, including seven tetrapeptides (YQLD, FYPE, YPEL, YKVP, YFYP, YLGY, YLEQ), a eleven-peptide (FSDIPNPIGSE) and a dodecapeptide (FSDIPNPIGSEN).

Generally, the antioxidant activity of peptides obtained by enzymatic hydrolysis is closely related to their amino acid constituents and sequences, which are also dependent on the hydrolysis degree and the proteases used. The presence of the hydrophobic amino acids and aromatic amino acids enhanced the oxidant quenching potential of peptides [26]. Peptides with valine (V) or leucine (L) at the N-terminus, or proline (P), histidine (H) or tyrosine (Tyr or Y) in the sequence, showed stronger antioxidant capacity [27]. Peptides containing Tyr and Tryptophan (Trp or W) strengthened antioxidant activity since the phenolic and indole groups in their structure could serve as hydrogen donors to inhibit free radical chain reactions; the antioxidant activity could be improved with Tyr and Trp at the terminus of the peptide chain, or when they were adjacent to each other [28]. Peptides derived from bovine β-casein and k-casein containing the sequences of KVLPVPEK and ARHPHPHLSFM were also reported with in vitro antioxidant property [29]. In this study, most of the peptides listed in Table 2 contain phenylalanine (Phe, F), Tyr and Trp, especially Phe and Tyr are mostly located at the N-terminus of the peptides. Some peptides such as YFYP and FYPE contain multiple aromatic amino acids. Furthermore, YPEL and YLEQ contain "Glu-Leu" structure, indicating potential antioxidant activity of the peptides [30]. These potentially antioxidant peptides in Table 2 were synthesized for the following analyses.

### Evaluation of DPPH Scavenging Ability and Cell Viability

**Fig. 3:**
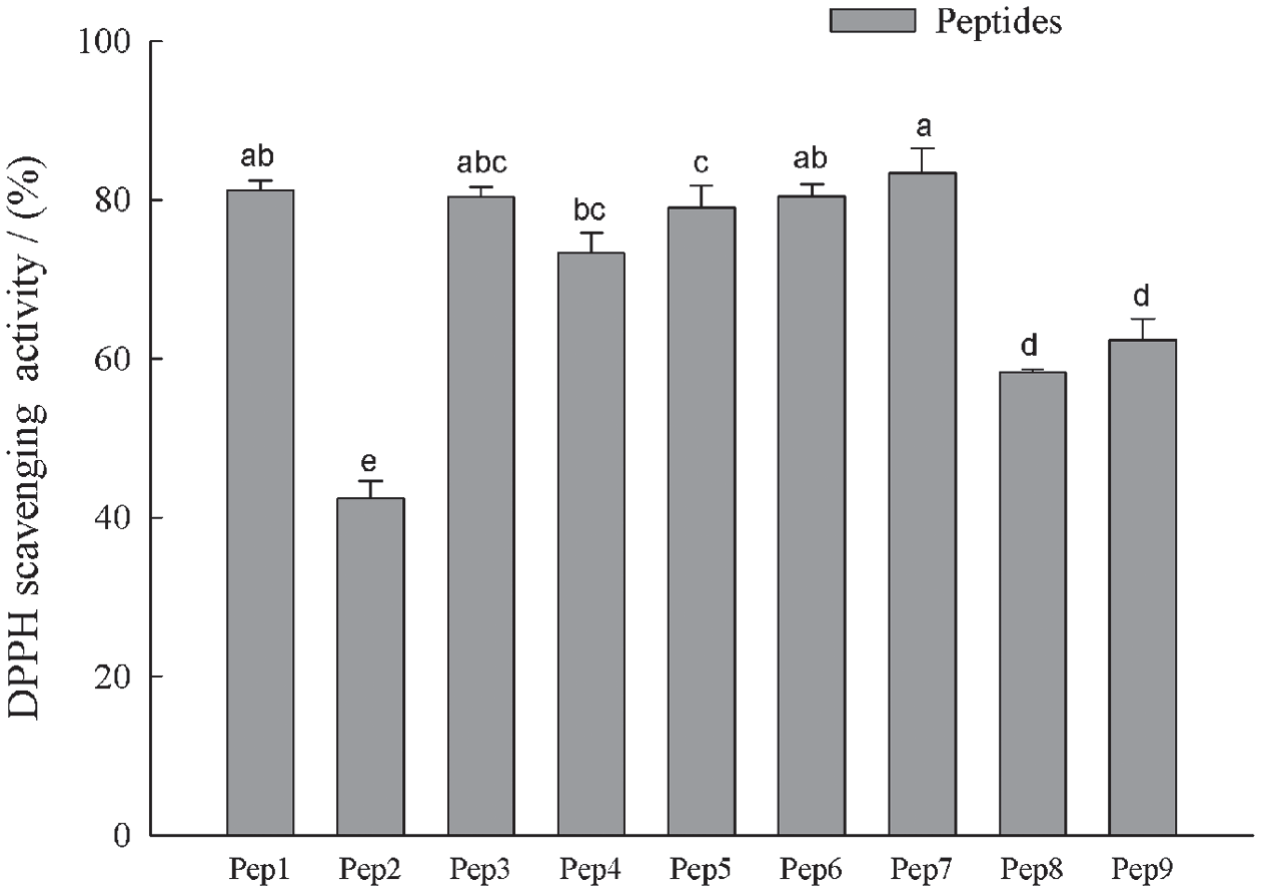
DPPH scavenging ability of nine synthesized peptides at concentration of 5.0 mg/ml. Data are presented as mean ± SD, and results marked with the same letters are not significantly different (*p* > 0.05).

Among the nine peptides, Pep7 showed the highest DPPH scavenging capacity, followed by Pep1, Pep6, and Pep 3 (Fig. 3) . These peptides were further selected for antioxidant evaluation.

**Fig. 4:**
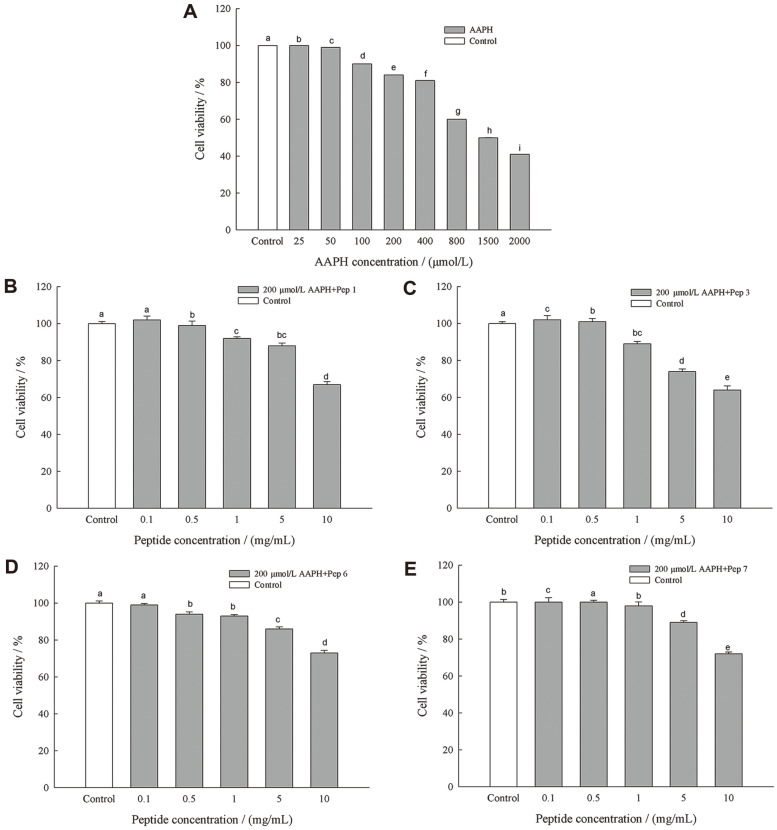
Effect of AAPH on the viability of HepG2 cells (A), and effects of Pep1(B), Pep3(C), Pep6(D), Pep7(E) on the viability of HepG2 cells. Data are presented as mean ± SD, and results marked with the same letters are not significantly different (*p* > 0.05).

Cell viability is an important indicator of cell survival or death when treated in condition of oxidative stress involving multiple pathways and mechanisms [31]. In this study, AAPH was used as a free radical initiator to induce cell oxidative stress damage. As shown in Fig. 4A, The viability of HepG2 cells was reduced as the AAPH concentration increased, and significantly decreased cell survival rate was observed at 200 μmol/l of AAPH. Therefore, AAPH at 200/L μmol that could cause obvious oxidative damage to the cells was selected for the following analysis. As shown in Figs. 4B-4E, the four peptides at lower concentrations (< 1.0 mg/ml) exhibited varying degrees of protection for the viability of HepG2 cells (above 90%) under the oxidant stress of AAPH (200 μmol/l). Higher concentration of the peptides (> 1.0 mg/ml) significantly (*p* < 0.05) decreased the cell viability, and it decreased to less than 80% for all the peptides at 10 mg/ml. These results indicated that selection of appropriate peptide concentration was important to avoid HepG2 cell damage. In the following experiments, the protective effect of these 4 peptides on the oxidative damage in the cells was studied at the concentrations of 0.1, 0.5, and 1.0 mg/ml.

### Genes and Protein Expression in Keap1-Nrf2 Pathway

**Fig. 5:**
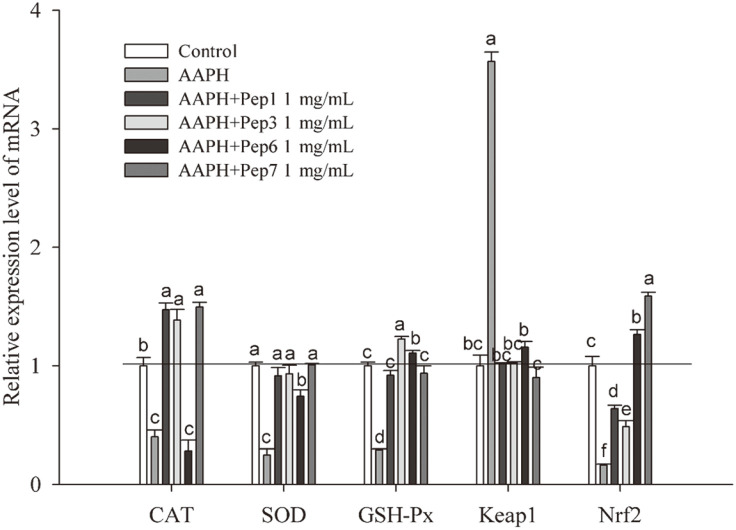
The relative mRNA expression level of CAT, SOD, GSH-Px, Keap1, and Nrf2. Each mRNA expression was normalized to that of the ribosomal protein GAPDH and expressed relative to the control level. Data are expressed as mean ± SD, and results marked with the same letters are not significantly different (*p* > 0.05).

Gene expression of the antioxidant enzymes (CAT, SOD, and GSH-Px), Keap1 and Nrf2 in HepG2 cells as affected by the antioxidant peptides (Pep1, Pep3, Pep6, and Pep7) was shown in Fig. 5. All peptides increased the mRNA expression of CAT, SOD, GSH-Px and Nrf2 to different extents, but decreased the mRNA expression of Keap1 as compared with the AAPH group, demonstrating basically the intracellular antioxidant effects of the four peptides. Pep7 showed similar degree of up-regulation capacity on the mRNA expression of SOD and GSH-Px, and higher degree of up-regulation capacity on the mRNA expression of CAT and Nrf2 when compared to the control group. However, Pep3 was most effective to increase the mRNA expression of GSH-Px, but Pep6 was least effective on the mRNA expression of CAT, indicating the significant effect of the terminal asparagine (Asn, N) in Pep3. There was one more amino acid (Asn) at the terminal of Pep3 compared to Pep6. Thus, the regulatory effect of the peptides on gene expression related to antioxidation in HepG2 cells varied with their sequence constituents.

**Fig. 6:**
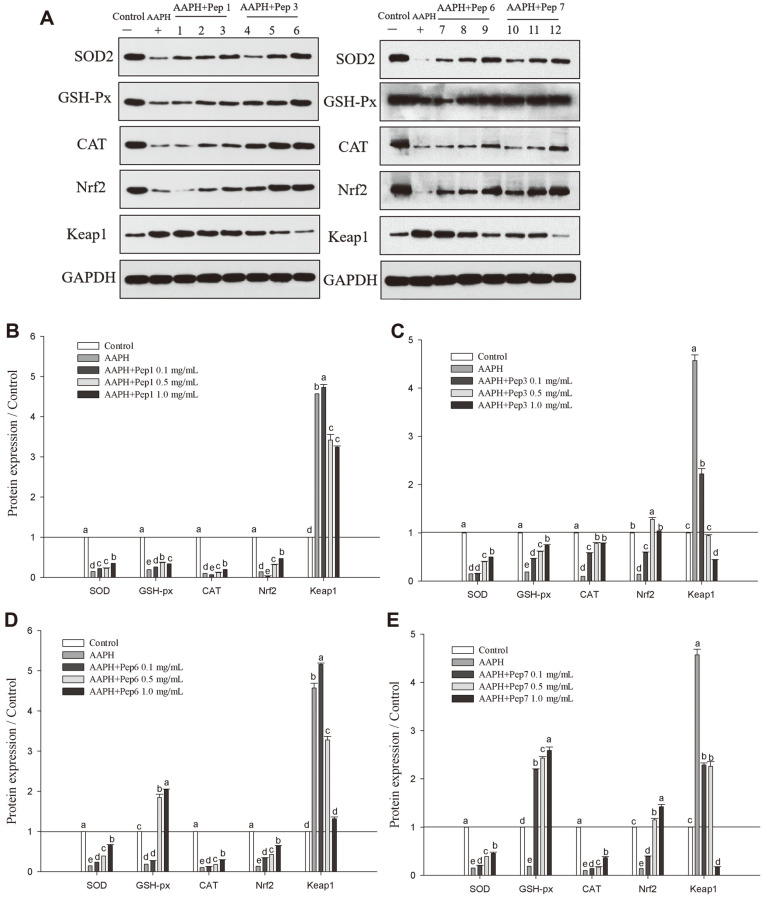
Protein expression related to antioxidant signaling pathway (A), and relative protein content in the presence of Pep1(B), Pep3(C), Pep6(D), and Pep7(E), when compared to the control. Lanes 1, 4, 7, 10 represent samples with Pep1, Pep3, Pep6, Pep7 at concentration of 0.1 mg/ml, respectively; Lanes 2, 5, 8, 11 represent samples with Pep1, Pep3, Pep6, Pep7 at concentration of 0.5 mg/ml, respectively; Lanes 3, 6, 9, 12 represent samples with Pep1, Pep3, Pep6, Pep7 at concentration of 1.0 mg/ml, respectively. Data are expressed as mean ± SD, and results marked with the same letters are not significantly different (*p* > 0.05).

Protein expression of CAT, SOD, GSH-Px, Keap1 and Nrf2 in HepG2 cells as affected by the antioxidant peptides (Pep1, Pep3, Pep6, and Pep7) was shown in Fig. 6. The expressed protein levels of SOD, GSH-Px, CAT, and Nrf2 in HepG2 cells treated with AAPH were significantly reduced compared to the control group and the groups treated with the peptides, indicating that treatment of the cells with each of the four peptides played a protective role in oxidative stress response. Among the four peptides, Pep3 and Pep7 demonstrated the strongest stimulative effect on the expression of Nrf2 and inhibitory effect on the expression of Keap1. Increased Nrf2 protein expression could protect the cells from damage under oxidative stress by stimulating the antioxidant enzymes system in the cells [32]. As shown in Figs. 6D and 6E, with increase of the peptide concentration from 0.1 to 1 mg/ml, the protein expression of SOD, GSH-px, CAT, Nrf2 increased and protein expression of Keap1 decreased to different levels in the presence of pep 6 and pep7, indicating the dose-dependent changes of antioxidant activities of the peptides. While, in the presence of pep 1 and pep 3, their antioxidant activities were also shown to be dose-dependent at the peptide concentration of 0.1-0.5 mg/ml (Figs. 6A and 6B). At the peptide concentration of 1 mg/ml, the protein expression of GSH-px in the presence of pep 1, and protein expression of Nrf2 in the presence of pep 3 decreased. However, some bioactive peptides were reported to be beneficial at low doses, while high doses of the same peptides were harmful [32, 33]. Yi *et al*. [34] and Efthalia *et al*. [35] reported that higher protein expression related to Keap1-Nrf2 pathway was not positively correlated with the peptide concentration. Thus dose-dependent manner of regulating target genes varied with the types of the peptides.

Activation of Keap1-Nrf2 system was not only influenced by the interaction with oxidants and electrophilic molecules, but also the action of specific peptides [36]. Therefore the antioxidant activities of the four peptides of this study were mediated by regulating modulatory genes and up-regulating the protein expression of antioxidant enzymes in HepG2 cells, and among of the four peptides, Pep 7 was the most active. Previous studies showed that casein hydrolysates significantly reduced oxidative damage via different molecular mechanisms. Caseinophosphopeptides and casein hydrolyzates were shown to bind prooxidant metals to inhibit lipid oxidation in cooked ground beef [37]. Liver antioxidant enzymes activities were significantly improved by casein hydrolysates with combined papain-flavourzyme treatment through enhancing Nrf2 transcription. Furthermore, 18 peptides were obtained as potential bioactive peptides and the dipeptide WM might inhibit Keap1-Nrf2 interaction as potential Nrf2 activators [38].

### Activities of Antioxidant Enzymes in HepG2 Cells Treated with Antioxidant Peptides

**Fig. 7:**
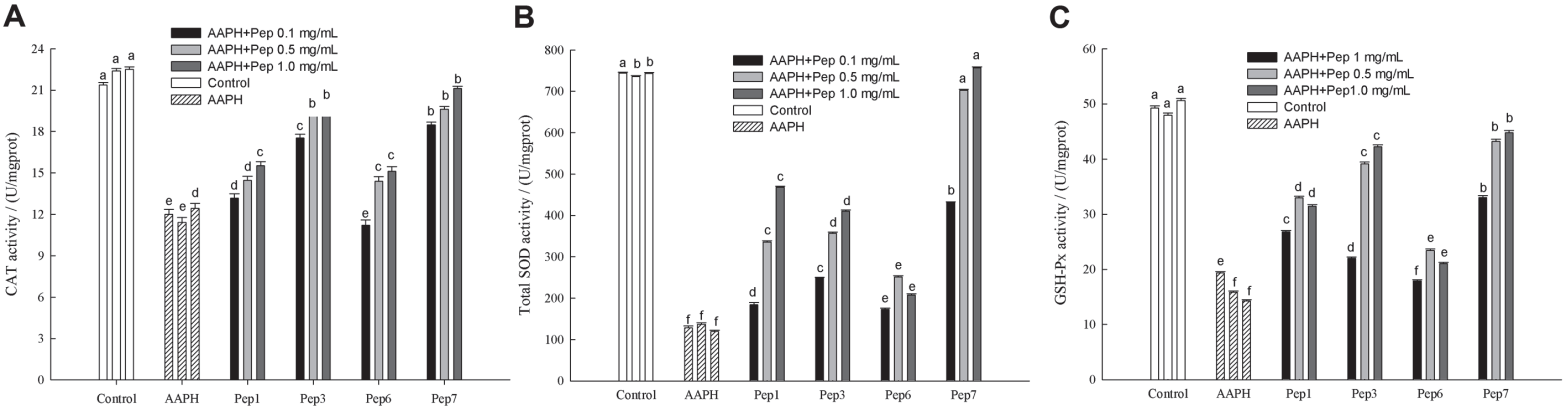
Effect of four peptides on the activities of CAT (A), SOD (B), and GSH-Px (C). Data are expressed as mean ± SD, and results marked with the same letters are not significantly different (*p* > 0.05).

Antioxidant responses can also be defined as the induction of antioxidant enzymes in the process mediated by the Keap1-Nrf2 system [39]. Among all the antioxidant enzymes, superoxide dismutase (SOD) is an antioxidant metal enzyme that can catalyze the disproportionation of superoxide anion radicals to generate oxygen and hydrogen peroxide; Catalase (CAT) is a resident enzyme of peroxisomes, decomposing hydrogen oxide into oxygen and water [40]; Glutathione peroxidase (GSH-Px) is an important peroxide decomposing enzyme widely present in the body [41]. Fig. 7 showed that the 4 antioxidant peptides could increase the activities of the intracellular SOD, CAT, and GSH-Px to different extents, indicating different antioxidant effects of the peptides in HepG2 cells. This could be explained by the different ability of the peptides to reduce expression of Keap1 that inhibited combination of Keap1 and Nrf2, leading to the Nrf2 transcription, a crucial process for regulating antioxidant enzymes including SOD, GSH-Px and CAT [42]. The activities of these enzymes tended to increase when the peptide concentration increased from 0.1 mg/ml to 1 mg/mL, matching the western blot analysis (Fig. 6). Particularly in cells treated with Pep7, the activities of SOD, CAT, and GSH-PX were significant (*p* < 0.05) higher than those of the AAPH group, and the groups treated by Pep1, Pep3, Pep6. Furthermore, the SOD activity in cells treated with Pep7 at 0.5 mg/ml reached the level of control group. Previously, casein-derived peptides were also shown to protect against oxidative stress in Caco-2 cell model by increasing the activities of intracellular antioxidant enzymes [36]. Besides, peptides could reveal their antioxidant effects by regulating activities of the antioxidant enzymes through the Keap1-Nrf2 system [43].

### ROS, MDA and GSH Production in HepG2 Cells Treated with Antioxidant Peptides

**Fig. 8:**
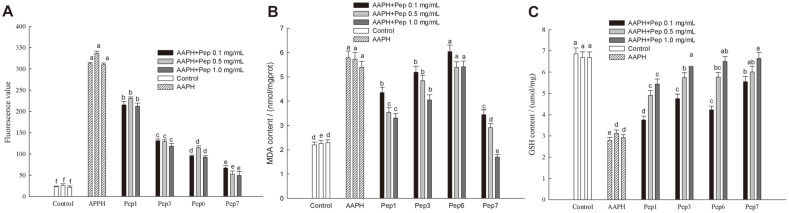
Effect of four peptides on the production of ROS (A), MDA (B), and GSH (C). Data are expressed as mean ± SD, and results marked with the same letters are not significantly different (*p* > 0.05).

To further define the antioxidant action mechanism, the 4 antioxidant peptides were evaluated for their effects on reactive oxygen species (ROS), malondialdehyde (MDA), and glutathione (GSH) contents. As a kind of reactive chemicals containing oxygen, ROS is formed as a natural by-product of normal metabolism of oxygen that plays an important role in cell signal transduction and homeostasis [44]. MDA is an important parameter indicating antioxidant capacity by reflecting the rate and intensity of lipid peroxidation in the body [45]. As shown in Fig. 8, addition of different concentrations of the peptides significantly (*p* < 0.05) inhibited the contents of ROS and MDA in HepG2 cells, and higher concentrations of the peptides exhibited more obvious effects. Both Pep1 and Pep3 had inhibitory effects on the production of MDA and ROS. However, Pep6 had more effect on ROS production, but no significant (*p* > 0.05) effect on MDA. Pep7 had significant (*p* < 0.05) effect on reduction of both ROS and MDA, and the levels of reduction was closer to those in the control group, indicating good antioxidant activity of Pep7.

GSH is a tripeptide containing a γ-amide bond and a sulfhydryl group, which can maintain normal immune system function with antioxidant effects by scavenging ROS [46, 47]. The content of GSH increased when each of the four peptides was present, and Pep 7 had a more positive effect on the increase of GSH. Thus, significant reduction of ROS, MDA formation and upregulation of GSH in HepG2 cells after treatment with Pep7 suggested an important role for peptide YFYP in protecting HepG2 cells against oxidative stress. However, there was no significant (*p* > 0.05) difference in GSH content between the AAPH group and Pep1 at 0.1 mg/ml, indicating uneffectiveness of Pep 1 at low concentration in achieving antioxidant activity by GSH increase.

Combining the above results of antioxidant tests and evaluation with HepG2 cells of this study, Pep7 (YFYP) was shown to be the most potent antioxidant peptide derived from casein by enzymatic hydrolysis with P-NY10 and P-2SD. Previously, YFYP showed higher antioxidant activity based on the percentage of lipid peroxidation when compared to the original peptide YFYPEL [48]. YFYPEL, which was derived from α_s1_-casein with pepsin or the proteases from *Arsukibacterium ikkense*, was reported to possess both antioxidant and ACE inhibitory activities [49]. YFYPE, which had glutamic acid (Glu or E) at C-terminus, was assumed to have antioxidant activity for the presence of Glu that contributed to the radical scavenging activity [19]. The high antioxidant activity of YFYP was attributed to the presence of proline at C-terminus, assuring the integrality of the peptide after gastrointestinal digestion as proline-containing peptides were resistant to intestinal proteolysis [50]. Other proline-containing peptide such as VLPVPQK derived from buffalo milk casein was shown to protect osteoblast cells against hydrogen peroxide (H_2_O_2_)-induced dysfunction and oxidative damage by increasing SOD and CAT activities [51]. Regarding Pep1 (YQLD) of this study, it was reported to be an antioxidant peptide due to the presence of Tyr known to be a radical scavenger [49]. Although APSFSDIPNPIGSENSE was reported to be an antioxidant peptide [52], the antioxidant activity of Pep3 (FSDIPNPIGSEN) and Pep6 (FSDIPNPIGSE) as characterized through Keap1-Nrf2 pathway in HepG2 cells of this study was not reported earlier.

The four antioxidant peptides obtained in this study could be potentially applied in functional foods or medical field. Early reports stated that the digestive proteases could hydrolyze proteins to release peptides with different lengths and free amino acids, and the exopeptidases and endopeptidases in intestinal epithelial cells further hydrolyzed oligopeptide fragments produced by pepsin and trypsin [53]. However, in vivo studies to investigate and track the metabolic fate of the bioactive proteins were still rare and sometimes produced controversial results [54]. Nevertheless, it was reported that the most typical well-known bioactive peptides with fragment lengths from 2 to 14 amino acids were able to easily pass through the gut tract [55]. Therefore, the digestion process of the bioactive peptides in this study needs further verification before their wide use in the fields of food or pharmaceuticals.

## Conclusions

Hydrolysis of casein by combination of the microbial P-NY10 and P-2SD (4:2) resulted in the hydrolysate (< 1 kDa) with good antioxident activity. Main antioxidant peptides with DPPH scavenging ability were identified from the hydrolysate as YQLD, FSDIPNPIGSEN, FSDIPNPIGSE, and YFYP, which were chemically synthesized for characterization in an oxidatively damaged HepG2 cell model induced by AAPH. These 4 peptides were found to decrease the cell oxidative damage to different extents by activating the Keap1-Nrf2 signaling pathway, leading to overexpression of the antioxidant enzymes such as SOD, CAT, and GSH-Px. Therefore, the 4 casein-derived antioxidant peptides obtained by using microbial proteases could serve as functional food ingredients for health promotion.

## Figures and Tables

**Table 1:** Primer sequences.

Gene	Primer name	Primer sequences (5' to 3')	Fragment size (bp)
SOD	H-SOD-RT-F	TggAgATAATACAgCAggCT	115
	H-SOD-RT-R	AgTCACATTgCCCAAgTCTC	
CAT	H-CAT-RT-F	CCTTCGACCCAAGCAA	97
	H-CAT-RT-R	CGATGGCGGTGAGTGT	
GPx1	H-GPx1-RT-F	AgAAgTgCgAggTgAACggT	172
	H-GPx1-RT-R	CCCACCAggAACTTCTCAAA	
Nrf2	H-Nrf2-RT-F	AgTgTggAgAggTATgAgCC	172
	H-Nrf2-RT-R	CgTTCCTCTCTgggTAgTAA	
Keap1	H-Keap1-RT-F	AGAGCGGGATGAGTGGCA	126
	H-Keap1-RT-R	GCTGAATTAAGGCGGTTTGTC	
Gapdh	H-Gapdh-RT-F	GGTGGTCTCCTCTGACTTCAACA	185
	H-Gapdh-RT-R	GTTGCTGTAGCCAAATTCGTTGC	

**Table 2:** The amino acid sequence of main peptides with molecular less than 1 kDa.

Peptide No.	Sequence	Length	Molecular weight	Source	Start	End	Intensity
Pep1	YQLD	4	537.2435	α_s1_-casein	169	172	1.99 × 10^10^
Pep2	FYPE	4	554.2377	α_s1_-casein	160	163	1.16 × 10^10^
Pep3	FSDIPNPIGSEN	12	1288.594	α_s1_-casein	194	205	6.33 × 10^9^
Pep4	YPEL	4	520.2533	α_s1_-casein	161	164	4.01 × 10^9^
Pep5	YKVP	4	505.29	α_s1_-casein	119	122	2.92 × 10^9^
Pep6	FSDIPNPIGSE	11	1174.551	α_s1_-casein	194	204	2.16 × 10^9^
Pep7	YFYP	4	588.2584	α_s1_-casein	159	162	1.29 × 10^9^
Pep8	YLGY	4	514.2428	α_s1_-casein	106	109	9.07 × 10^9^
Pep9	YLEQ	4	551.2591	α_s1_-casein	109	112	6.89 × 10^8^
